# Primary care during the transition to adult care for adolescents involved with pediatric specialty services: a scoping review protocol

**DOI:** 10.1186/s13643-021-01593-w

**Published:** 2021-02-02

**Authors:** Kyleigh Schraeder, Brooke Allemang, Cathie Scott, Kerry McBrien, Gina Dimitropoulos, Ashley Felske, Susan Samuel

**Affiliations:** 1 Department of Pediatrics, Cumming School of Medicine, University of Calgary, Calgary, Alberta Canada; 2 Faculty of Social Work, University of Calgary, Calgary, Alberta Canada; 3 PolicyWise for Children & Families, Calgary, Alberta Canada; 4 Department of Community Health Sciences, Cumming School of Medicine, University of Calgary, Calgary, Alberta Canada; 5 Department of Family Medicine, Cumming School of Medicine, University of Calgary, Calgary, Alberta Canada

**Keywords:** Transition to Adult care, Adolescent, Young adult, Adolescent health, Adolescent health services, Pediatrics, Primary care physicians, Primary health care

## Abstract

**Background:**

Of the 15–20% of youth in North America affected by a chronic health condition (e.g., type 1 diabetes, cystic fibrosis) and/or mental health or neurodevelopmental disorder (e.g., depression, eating disorder, Attention Deficit-Hyperactivity Disorder), many often require lifelong specialist healthcare services. Ongoing primary care during childhood and into young adulthood is recommended by best practice guidelines. To date, it is largely unknown if, how, and when primary care physicians (PCPs; such as family physicians) collaborate with specialists as AYAs leave pediatric-oriented services. The proposed scoping review will synthesize the available literature on the roles of PCPs for AYAs with chronic conditions leaving pediatric specialty care and identify potential benefits and challenges of maintaining PCP involvement during transition.

**Methods:**

Arksey and O’Malley’s original scoping review framework will be utilized with guidance from Levac and colleagues and the Joanna Briggs Institute. A search of databases including MEDLINE (OVID), EMBASE, PsycINFO, and CINAHL will be conducted following the development of a strategic search strategy. Eligible studies will (i) be published in English from January 2004 onwards, (ii) focus on AYAs (ages 12–25) with a chronic condition(s) who have received specialist services during childhood, and (iii) include relevant findings about the roles of PCPs during transition to adult services. A data extraction tool will be developed and piloted on a subset of studies. Both quantitative and qualitative data will be synthesized.

**Discussion:**

Key themes about the roles of PCPs for AYAs involved with specialist services will be identified through this review. Findings will inform the development and evaluation of a primary-care based intervention to improve transition care for AYAs with chronic conditions.

**Supplementary Information:**

The online version contains supplementary material available at 10.1186/s13643-021-01593-w.

## Introduction

Between 15 and 20% of youth in North America are affected by a chronic health condition (e.g., type 1 diabetes, cystic fibrosis, asthma) and/or mental health or neurodevelopmental disorder (e.g., depression, eating disorder, attention deficit-hyperactivity disorder (ADHD), autism spectrum disorder [1–3]). Many of these adolescents and young adults [AYA] require lifelong specialist healthcare or mental health services, defined herein as services provided by physicians with additional training and expertise in a defined area [1, 2, 4]. AYA are typically referred to pediatric specialist services in childhood or adolescence by their primary care physician (PCP), such as a family physician, who plays a key role in the initial assessment and identification of issues [5, 6]. During and following specialist involvement, it is recommended that AYA continue to receive primary care to meet their general healthcare needs (e.g., vaccinations, contraceptives [7–10]). Further, PCPs can provide AYA and their families with an ongoing patient-provider relationship, which may be beneficial during potential gaps in services as youth become older [7]. For example, during the transition from pediatric- to adult-oriented services, continuous primary care is associated with fewer hospitalizations for certain AYA populations (e.g., diabetes [11], severe mental illness [12]). Despite transition best practice guidelines [1, 9] and acknowledgement by clinicians, researchers, and policy-makers about the importance of continuous primary care during the transition to adult care [13], it is unclear how PCPs are involved in caring for adolescents (ages 13–18) and young adults (ages 19–25) leaving specialist pediatric services [14].

**Fig. 1:**
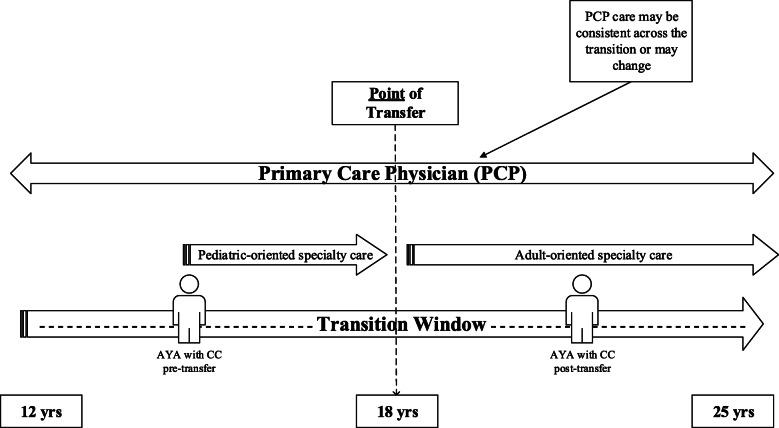
Transition window. Point of transfer is defined as the point when an adolescent or young adult (AYA) with a chronic condition (CC) moves from one provider to another (i.e., actual point when an AYA moves from a pediatric to an adult-oriented care provider [1]). This is typical around age 18; however, this age can vary. The transition window refers to the entire period of time when processes related to preparing and planning for and adjusting to transition typically occur [1]. Adult specialist care is possible at any point following transfer. This study defines primary care as any services provided by a primary care physician (PCP; e.g., family physicians, nurse practitioners, etc., and in some jurisdiction, pediatricians) to patients across the lifespan

Transition has been commonly described as “the purposeful, planned *process* that addresses the medical, psychosocial, and educational/vocational needs of young people and young adults with chronic physical and medical conditions as they move from child-centered to adult-oriented healthcare systems” [15]. This ongoing process is recommended to begin at age 12 and often involves multiple providers, includes self-management skill building, vocational planning, and the provision of adolescent-specific health information [7–9]. The transfer between pediatric and adult specialists typically occurs at 18 years old, though this may vary across jurisdictions [16, 17]. Transfers between providers can pose substantial risks for AYAs (e.g., delays in needed treatment, disengagement from services [15, 18–21]). Yet, most of the research in this area (e.g., [22–24] has focused on addressing barriers to successful service transfers [25, 26], and not how PCPs are involved [27, 28]. As depicted in Fig. 1, some AYAs may maintain relationships in primary care, while others may be discharged to a new PCP after specialist services. Little is known, however, about how many AYAs receive primary care before and after transfer. This information is needed to address key challenges associated with care coordination during transfers in care and transition, among providers in different settings and systems of care (e.g., primary care, specialty care).

A recent systematic review of pediatric transition interventions identified only three published studies with a primary care component [29]. In two of the studies reviewed, both based in hospital settings, a case manager facilitated appointments with PCPs and primary care outcomes were not reported (e.g., AYA engagement with primary care [30, 31]). The third study involved a quality improvement educational initiative across five academic primary care centers in the USA [32]; the main focus, and outcome, of this study was on the development of practice-wide transition policies. Limitations of this review included a narrow focus on studies involving older AYAs (> 16 years old), and studies with an intervention or evaluation component. Research on primary care for younger AYAs (ages 12–16) with chronic conditions can inform models of transition care. Further, non-intervention based research can help us to understand optimal PCP roles during transition. A broader review of the available primary care literature, including research with younger AYA and qualitative studies with AYA, caregivers, and clinicians, is needed to inform clinical practice or policy change relevant to transition care.

Literature on the patient-centered medical home model, first established in pediatrics for AYA with chronic conditions [9, 33, 34], is relevant to the topic of AYA healthcare transitions [35, 36]. In primary care, the medical home [37] represents a vision of practice emphasizing the roles of PCPs (namely family physicians) in delivering high-quality, coordinated, continuous, family-centered, compassionate, and comprehensive care [1] across the lifespan. Recently, the “medical neighborhood” was developed to recognize the importance of establishing and maintaining links between PCPs and providers in other settings (e.g., hospitals, community agencies [1, 38, 39]). However, the nature of collaborations between PCPs and specialists (or sub-specialists) in the ‘medical neighborhood’ or within the ‘medical home’ itself (e.g., co-location, shared-care models), for AYAs with chronic conditions has not been adequately studied [40, 41]. Specifically, how PCPs collaborate or consult with specialists for AYAs throughout the transition period is poorly understood [42, 43]. To address this gap, a description of collaborative care models (primary-specialty care) that aim to facilitate the transition process is needed.

In order to synthesize the available published literature and identify knowledge gaps on the topic of primary care and transitions from pediatric to adult care, a scoping review will be conducted [44, 45]. The overall aim of this review is to summarize the evidence about how PCPs support AYA (i.e., 12–25 years old) during the transition period. Additional sub-questions include (1) how many AYAs visit PCPs before and after transfer?; (2) What are the potential benefits or challenges of PCP involvement during transition?; (3) What models of collaborative primary-specialty care models exist for transition care? The ultimate goal of this work will be to inform the development of a Canadian-based primary-specialty care intervention to optimize care transitions for AYAs.

## Method

The review protocol has been registered within the Open Science Framework database (registration DOI: 10.17605/OSF.IO/6X4MQ). The proposed scoping review will be reported in accordance with the reporting guidance provided in the Preferred Reporting Items for Systematic Reviews and Meta-analyses (PRISMA) extension for Scoping Reviews (PRISMA-ScR) [46] (see checklist in Additional file 1). Any amendments made to this protocol when conducting the study will be outlined in the Open Science Framework and reported in the final manuscript.

### Scoping review framework

Arksey and O’Malley [47] published the first rigorous methodological framework for conducting scoping reviews, which includes six steps: (1) identifying the research question; (2) identifying relevant studies; (3) selecting studies; (4) charting the data; (5) collating, summarizing and reporting the results; and (6) consulting with relevant stakeholders. Levac, Colquhoun, and O’Brien [48] advanced Arksey and O’Malley’s [47] framework by providing further recommendations for each of these six steps. Peters and colleagues [44] of the Joanna Briggs Institute (JBI) and Joanna Briggs Collaborating Centers [49] offer the most recently updated guidelines for conducting scoping reviews, having built upon Arksey and O’Malley [47] and Levac and colleagues’ [48] work.

The proposed scoping review will follow the framework originally developed by Arksey and O’Malley [47], and the enhancements published by Levac et al. [48] and Peters et al. [44, 49]. Given the nature of a scoping review, ethical approval will not be sought.

#### Step 1: identifying the research questions

The aforementioned research questions were developed through an iterative process with input from our research team.

#### Step 2: identifying relevant studies

A systematic online search strategy will be developed, with the assistance of an experienced librarian at the Alberta Children’s Hospital (Alberta, Canada), to identify relevant peer-reviewed articles in online databases: MEDLINE (OVID), EMBASE, PsycINFO, and Cumulative Index to Nursing and Allied Health Literature (CINAHL). A list of anticipated search terms and an example search string are provided in Additional file 2; terms have been compared against Medical Subject Headings (MeSH) to insure relevance and breadth of search results. An additional screening technique, referred to as the snowball method [50], will be applied to the reference lists of studies identified through the initial database search to identify additional studies of relevance.

#### Step 3: selecting studies

##### Study screening

Study screening will be completed at two levels. First, two independent reviewers will screen titles and abstracts obtained from the database search for inclusion eligibility, as described below. If an article’s relevance is unclear from the title or abstract, the article will be kept for further review in level two. The second level of screening will involve two independent reviewers reviewing full-text articles for inclusion. Any disagreements will be resolved via group discussion to achieve consensus. Microsoft Excel will be used to manage retrieved titles and abstracts, and will enable us to (i) identify and remove duplicates; (ii) perform, manage, and document the screening process of titles and abstracts; (iii) categorize publications based on their inclusion or exclusion; and (iv) track memos and notes by the research team related to screening decisions. Finally, a study inclusion flowchart, following PRISMA guidelines, will be created.

##### Inclusion and exclusion criteria

Inclusion criteria were developed following JBI guidelines [49] and categorized by (i) types of studies (e.g., study year, design), (ii) population (e.g., participant age, diagnoses), (iii) context (e.g., study country of origin, language), and (iv) concept (e.g., relevant study findings).

(i) Type of study

Type of study refers to characteristics of the sources of information or articles, included. Studies published from January 2004 onwards will be included; this timeframe aligns with the years when the American Academy of Family Physicians adopted the primary care ‘medical home’ model, and when research on this topic increased worldwide [51–53]. Only primary research studies will be included; case studies, reports, congresses, clinical practice guidelines, theses, and opinion-driven reports (e.g., editorials, literature/narrative reviews) will be excluded. Protocol studies, or studies describing a model or treatment without an evaluation component, will be excluded; otherwise, there will be no limitations on study design (e.g., naturalistic observational study, retrospective cohort study, mixed-methods study, qualitative study).

(ii) Population

The population refers to participant characteristics that are of interest and will be relevant to address the proposed research questions [49]. For this review, included studies will have a clear population focus on AYAs, defined as ≥ 50% adolescents (ages 12 to 18) and/or young adults (19 to 25) in the participant sample. This age criterion will ensure study findings are relevant to inform transition practices for AYAs transferring from pediatric- to adult-oriented care; studies focusing only on children (< 12 years old), or adults (> 25 years old), will be excluded. To be included, AYA participants must have at least one identified physical health and/or mental health condition, which is chronic in nature; broadly defined as: a condition that lasts ≥ 3 months, is not (yet) curable, affects an AYA’s daily activities, and requires ongoing medical and/or psychological care [3, 54]. A broad range of medical, neurodevelopmental, and psychological conditions, that are typically chronic in nature, will therefore be included (see Additional file 2 for a complete list of diagnoses/problems from our search strategy). Participants described as “at-risk” for a chronic condition, but who have not yet been identified or diagnosed (e.g., screening for substance use) will not meet our population criteria. Finally, AYA participants must be receiving, or have received, specialty health and/or mental health services at some point in childhood or adolescence (0–18 years old); specialty care is explicitly defined under the “Concept” section.

(iii) Context

For the purposes of our scoping review, we will not limit studies based on geographical location or socio-cultural factors (e.g., gender, race, ethnicity). However, only studies available in English will be included. Studies will not be limited by healthcare setting or system-related factors (e.g., physician payment models).

(iv) Concept

Concept characteristics typically act as points of guidance when extracting relevant findings and map onto the outcomes of interest [49]. In this review, a key concept will be the intersection between primary care and specialty (secondary or tertiary) care for AYAs, which must be clearly described in the article. This concept may include reference to how primary and specialty care providers communicate, collaborate, or co-manage the care of AYA with chronic conditions. It may also involve discussion of the responsibilities of primary or specialty care providers during the transition from pediatric specialty care. Primary care will be defined as any services provided by a general healthcare provider or PCP who provides first point of contact care to patients across the lifespan, including family physicians and nurse practitioners. In some healthcare settings, pediatricians can serve as PCPs for AYAs up to a certain age (in other settings, pediatricians are specialists and typically require referral); we will consider articles with pediatricians as PCPs on a case-by-case basis for their relevance to our review.

Pediatric specialty care will be defined as any specialist health and/or mental health services provided to youth by a physician with additional training in a defined area (e.g., psychiatrist, rheumatologist, endocrinologist, oncologist). We acknowledge that some specialist services offered in the community may not involve specialist physicians (e.g., community-based mental health programs, addiction centers, school-based interventions for neurodevelopmental conditions); we will consider these articles if there is a clear link with primary care or PCPs, and for their relevance to understanding transition care for AYAs with chronic conditions. Articles focused on programs or services based exclusively in primary care (or the ‘medical home’) will be excluded (e.g., programs delivered in primary care by social workers, dieticians, nurses). Primary care-based models of care will only be considered if there is some mention of specialist physician involvement (e.g., co-location or consultation models), given the focus on understanding the integration of primary care and specialist or secondary-level care.

#### Step 4: charting and extracting the data

Following published recommendations [44, 49], a data extraction tool will be created to organize key data items (e.g., type of study, population, study context) and relevant study findings that map onto the proposed research aims/questions. This tool will be developed through an iterative process, including pilot testing and research team discussion and consultation. Additional file 3 outlines a possible template for this tool. Pilot testing will involve coding a random set of included articles using this template by at least three members of the research team (KS, AF, BA) to ensure accurate and consistent data extraction among three coders. Necessary revisions to the tool will be made before all included studies are coded. NVivo software [QSR International Pty Ltd. Version 12, 2018 [55]] will be used to track, organize, and complete the data extraction process.

#### Step 5: collating, summarizing, and reporting the results

The data extracted in step 4 will be summarized in a tabular format, see Additional file 3 [46]. We will first summarize the available evidence on the topic (e.g., types of studies included, clinical contexts, and AYA populations). Where appropriate, qualitative description [56, 57], using summative content analysis [58], will be conducted to count and compare content from the included quantitative, qualitative, and mixed methods studies relating to our research questions [59, 60]. Descriptive statistics, including percentages and frequencies, will be reported as they pertain to specific outcomes of interest and research sub-questions (e.g., percentage of AYA with PCP visits pre- and post-transfer, or percentage of AYA who experience improved health outcomes post-transfer). Benefits and challenges of PCP involvement during transition, as reported in survey-based and qualitative studies, will be summarized (e.g., attitudes/beliefs about challenges of PCP involvement). Steps will be taken to maximize validity and rigor of our analysis and summary, such as independent and team analysis of the extracted data to collaboratively develop and refine themes, re-reading included articles, reviewing the raw data and/or including direct quotes (if applicable), and comparing interpretations within our multi-disciplinary research team [60]. Reflexivity processes, such as attending to preconceptions brought into the project and memo-writing, will also account for researchers’ influences on the findings [61].

#### Step 6: consulting with relevant stakeholders

This protocol received input from all authors, who have expertise across disciplines: primary care (KS, KM, CS), pediatrics (BA, SS, GD), and mental health (KS, BA, GD). All authors are part of an existing pediatric transition research program, which includes key stakeholders and leaders in transition research, knowledge translation, and health policy in the province of Alberta, Canada. We plan to receive valuable feedback on the preliminary findings from this review at local and national research conferences.

### Discussion and dissemination

Findings from this scoping review will be important for three main reasons. First, this review will address the role of primary care for AYAs transitioning from pediatric specialist services, which is a topic that has not been adequately addressed in the literature. Specifically, we hope to clarify the roles of PCPs, and the perceived benefits and challenges of their involvement, for AYAs with chronic conditions during the transition period. Previous reviews in this area have demonstrated that no pediatric transition interventions to date have evaluated primary care outcomes. A broader scan of the literature is needed to identify evidence to clarify and support best practice guidelines [1, 9], which recommend that PCPs should be actively involved in the transition process. Additionally, clarity surrounding PCP roles in the care of AYA with chronic conditions will, ideally, prevent the duplication of services in primary care versus specialty care. Second, this review will clarify the literature on collaborative care models which include PCPs and specialists to support AYA with chronic conditions. Research suggests PCPs would prefer to build collaborative relationships with sub-specialists instead of simply transferring management of their referred patients to them [40]. However, various types of collaborative practices, specifically during the transition period, may exist and this review can help address this gap. Finally, PCPs need to be equipped to manage AYAs with chronic conditions given the increasing number of children with chronic conditions surviving into adulthood, and the inadequate number of adult specialists capable of providing this care [62, 63]. Thus, information on the proportion of AYAs supported by PCPs (and/or by specialists) is critical for informing resource allocation in primary care for this growing patient population.

Overall, the results of this review will be used to (1) clarify the available literature on the benefits and challenges of PCP involvement for AYAs transitioning from pediatric services, and (2) inform and develop a primary-specialist care intervention to improve transition care for this population. Potential limitations of the proposed scoping review include varying definitions of a “primary care provider” (e.g., general pediatricians, nurse practitioners) which may vary depending on where the study was conducted, as well as a lack of consensus of measurable transition outcomes in the literature. We are also limiting this review to English language articles given we cannot practically assess literature in other languages. Through our synthesis of the evidence, however, our findings will have the potential to guide researchers, clinicians, and policy-makers to address key gaps identified [45]. Results will be disseminated through publication in a peer-reviewed journal, as well as, through conference presentations at various local and international conferences related to AYA healthcare transitions (e.g., Chronic Illness and Disability Conference: Transition from Pediatric to Adult-Based Care; Youth Transitions to Adulthood) and primary care (e.g., North American Primary Care Research Group).

## Supplementary Information

**Additional file 1.** PRISMA-ScR (Preferred Reporting Items for Systematic review and Meta Analysis extension for Scoping Reviews) 2018 Checklist.

**Additional file 2.** Anticipated Key Terms Used for Search Strategy.

**Additional file 3.** Data Extraction Tool (Codebook).

## Data Availability

Data sharing not applicable to this protocol article as no datasets were generated or analyzed yet.

## Abbreviations

AYA: Adolescents and young adults
PCP: Primary care physician
