# Supplementary material for: Primary care during the transition to adult care for adolescents involved with pediatric specialty services: a scoping review protocol
Source: Syst Rev. 2021 Feb 2;10:46. doi: 10.1186/s13643-021-01593-w (PMC7856752; doi:10.1186/s13643-021-01593-w)
Supplement: Supplementary file 2 — Additional file 2. Anticipated Key Terms Used for Search Strategy. [file 13643_2021_1593_MOESM2_ESM.docx]

**Additional File 2.** Anticipated Key Terms Used for Search Strategy

| **Context/Concept** | **Patient Population** | **Childhood-onset chronic conditions** |
| --- | --- | --- |
| Primary Health Care Primary Care  Primary healthcare  General Practice  Physicians, Family  Physicians, Primary Care  Family practice  General practitioners  Medical home  Behavioral Medicine  Psychology  Psychiatry  Social Medicine  Community Medicine  Adolescent psychology  Child psychology  Clinical psychology  Medical psychology  Psycho-oncology  Adolescent psychiatry  Child psychiatry  Community psychiatry  Preventive psychiatry  Psychosomatic medicine | Adolescents (13-18 years)  Adolescence  Teenagers  Teens  Youth  Young adults (19-24 years)  Emerging adults | Chronic disease  Chronic illness  Pediatrics  Adolescent medicine  Child special healthcare needs  Chronic physical health conditions  Chronic pain  Diabetes  Asthma  Arthritis  Cancer  Celiac disease  Cerebral Palsy  Congenital heart defects  Cystic fibrosis  Epilepsy  Seizures  HIV  Acquired Immunodeficiency  Syndrome  IBD  Crohn disease  Migraines  Muscular dystrophy  Neoplasms  Anemia  Spinal dysraphism  Sickle cell  Spina bifida  Tumour  Transplants  Mental disorders [F03, all disorders listed under this MeSH term]  Mental health*  ADHD  Anxiety disorders  Anorexia  Bulimia Nervosa  Depression  Psychotic disorders  Childhood schizophrenia  Mood disorders  Learning disorders  Intellectual disability  Conduct disorder  Neurocognitive disorders  Sleep wake disorders  Insomnia  Sexual dysfunctions,  psychological  Substance-related disorders  Drug abuse  Addiction  Disruptive behaviour disorders  Trauma and stressor-related  disorders  Chronic pain  Alcohol-related disorders  Pain management  Community mental health services  Specialty pediatric care  Psychiatry |

**Example of Search String for PSYCH INFO Database**

1. primary health care/

2. general practitioners/ or family medicine/ or family physicians/

3. (primary care or primary health or family medicine or primary healthcare or general practi* or family practice* or family physician* or family doctor* or general practice physician*).ti.

4. (primary care or primary health or family medicine or primary healthcare or general practi* or family practice* or family physician* or family doctor* or general practice physician*).ab. /freq=2

5. or/1-4

6. exp chronic illness/ or exp chronic stress/ or exp "chronicity (disorders)"/ or exp mental disorders/ or exp physical disorders/

7. exp pediatrics/

8. adolescent health/ or adolescent behavior/

9. behavioral medicine/

10. exp psychology/

11. exp psychiatry/

12. (p?ediatric* or adolescent medicine or adolescent health or behavio?ral medicine or psychology* or psychiatry* or social medicine or community medicine).ti.

13. (p?ediatric* or adolescent medicine or adolescent health or behavio?ral medicine or psychology* or psychiatry* or social medicine or community medicine).ab. /freq=2

14. exp organ transplantation/

15. chronic pain/

16. exp diabetes mellitus/

17. exp arthritis/

18. asthma/

19. celiac disease/

20. cerebral palsy/

21. exp Heart Disorders/ and exp Congenital Disorders/

22. cystic fibrosis/

23. exp epilepsy/

24. exp seizures/

25. hemophilia/

26. exp hiv/

27. irritable bowel syndrome/

28. exp gastrointestinal disorders/

29. migraine headache/

30. muscular dystrophy/

31. exp neoplasms/

32. anemia/

33. exp Spina Bifida/

34. (chronic disease* or chronic illness* or transplant* or chronic pain or juvenile arthritis or arthri* or asthma or celiac* or cerebral palsy or congenital heart* or cystic fibrosis or diabete* or epileps* or seizure or hemophilia* or human immunodeficiency virus* or HIV* or AIDS* or inflammatory bowl disease* or IBD* or crohn* or ulcerative colitis or migraine* or muscular dystroph* or neoplasm* or cancer* or tumour* or anemia or sickle cell or spinal bifida).ti.

35. (chronic disease* or chronic illness* or transplant* or chronic pain or juvenile arthritis or arthri* or asthma or celiac* or cerebral palsy or congenital heart* or cystic fibrosis or diabete* or epileps* or seizure or hemophilia* or human immunodeficiency virus* or HIV* or AIDS* or inflammatory bowl disease* or IBD* or crohn* or ulcerative colitis or migraine* or muscular dystroph* or neoplasm* or cancer* or tumour* or anemia or sickle cell or spinal bifida).ab. /freq=2

36. exp Disorders/

37. exp mental disorders/

38. Conduct Disorder/

39. exp "Depression (Emotion)"/

40. exp Pain Management/ or exp Pain/

41. exp sexual function disturbances/

42. (pain management or psychological sexual dysfunctions or dyspareunia or erectile dysfunction or gender dysphoria or premature ejaculation or sexual disorder* or gender disorder* or vaginismus).ti.

43. (pain management or psychological sexual dysfunctions or dyspareunia or erectile dysfunction or gender dysphoria or premature ejaculation or sexual disorder* or gender disorder* or vaginismus).ab. /freq=2

44. (mental disorder* or mental illness* or anxi* or panic* or depress* or anorexia or bulimia or autis* or mood disorder* or attention deficit or hyperactiv* or ADHD or learning disorder* or intellectual disability* or disruptive mood or dysregulation disorder* or disruptive behavio?r or psycho* or schizophrenia or mental retardation or trauma or aggression or conduct or externali* or internali* or medical home*).ti.

45. (mental disorder* or mental illness* or anxi* or panic* or depress* or anorexia or bulimia or autis* or mood disorder* or attention deficit or hyperactiv* or ADHD or learning disorder* or intellectual disability* or disruptive mood or dysregulation disorder* or disruptive behavio?r or psycho* or schizophrenia or mental retardation or trauma or aggression or conduct or externali* or internali* or medical home*).ab. /freq=2

46. (substance-related disorder* or alcohol-related disorder* or alcohol-induced disorder* or alcohol amnestic disorder* or alcoholic korsakoff syndrome* or alcoholic neuropathy or alcoholic cardiomyopathy or fetal alcohol spectrum disorder* or alcoholic liver disease* or alcoholic fatty liver or alcoholic hepatitis or alcoholic liver cirrhosis or alcoholic pancreatitis or alcoholic psychoses or alcoholic intoxication or alcoholism or binge drinking or wernicke encephalopathy or amphetamine-related disorder* or cocaine-related disorder* or drug overdose* or inhalant abuse or marijuana abuse or neonatal abstinence syndrome* or opioid-related disorder* or heroin dependence or morphine dependence or opium dependence or phencyclidine abuse or substance-induced psychoses or substance abuse or substance withdrawal syndrome* or trauma related disorder* or stressor related disorder* or adjustment disorder* or traumatic stress disorders* or battered child syndrome* or combat disorder* or psychological trauma).ti.

47. (substance-related disorder* or alcohol-related disorder* or alcohol-induced disorder* or alcohol amnestic disorder* or alcoholic korsakoff syndrome* or alcoholic neuropathy or alcoholic cardiomyopathy or fetal alcohol spectrum disorder* or alcoholic liver disease* or alcoholic fatty liver or alcoholic hepatitis or alcoholic liver cirrhosis or alcoholic pancreatitis or alcoholic psychoses or alcoholic intoxication or alcoholism or binge drinking or wernicke encephalopathy or amphetamine-related disorder* or cocaine-related disorder* or drug overdose* or inhalant abuse or marijuana abuse or neonatal abstinence syndrome* or opioid-related disorder* or heroin dependence or morphine dependence or opium dependence or phencyclidine abuse or substance-induced psychoses or substance abuse or substance withdrawal syndrome* or trauma related disorder* or stressor related disorder* or adjustment disorder* or traumatic stress disorders* or battered child syndrome* or combat disorder* or psychological trauma).ab. /freq=2

48. (neurocognitive disorder* or amnesia or alcoholic korsakoff syndrome* or cognition disorder* or auditory perceptual disorders* or huntington disease* or cognitive dysfunction* or consciousness disorder* or delirium or dementia or alzheimer disease* or primary progressive aphasia or primary progressive nonfluent aphasia or creutzfeldt-jakob syndrome* or diffuse neurofibrillary tangles with calcification or frontotemporal lobar degeneration or "pick disease of the brain" or kluver-bucy syndrome* or lewy body disease* or acquired dyslexiaor or pure alexia or sleep wake disorder* or dyssomnias or sleep deprivation or sleep disorders* or jet lag syndrome* or "disorders of excessive somnolence" or idiopathic hypersomnolence or kleine-levin syndrome* or narcolepsy or cataplexy or nocturnal myoclonus syndrome* or restless legs syndrome* or sleep apnea syndrome* or sleep apnea or obesity hypoventilation syndrome* or "sleep initiation and maintenance disorders" or insomnia or parasomnias or nocturnal paroxysmal dystonia or rem sleep parasomnias or rem sleep behavior disorder* or sleep paralysis or sleep arousal disorder* or night terror* or somnambulism or sleep bruxism or sleep-wake transition disorder*).ti.

49. (neurocognitive disorder* or amnesia or alcoholic korsakoff syndrome* or cognition disorder* or auditory perceptual disorders* or huntington disease* or cognitive dysfunction* or consciousness disorder* or delirium or dementia or alzheimer disease* or primary progressive aphasia or primary progressive nonfluent aphasia or creutzfeldt-jakob syndrome* or diffuse neurofibrillary tangles with calcification or frontotemporal lobar degeneration or "pick disease of the brain" or kluver-bucy syndrome* or lewy body disease* or acquired dyslexiaor or pure alexia or sleep wake disorder* or dyssomnias or sleep deprivation or sleep disorders* or jet lag syndrome* or "disorders of excessive somnolence" or idiopathic hypersomnolence or kleine-levin syndrome* or narcolepsy or cataplexy or nocturnal myoclonus syndrome* or restless legs syndrome* or sleep apnea syndrome* or sleep apnea or obesity hypoventilation syndrome* or "sleep initiation and maintenance disorders" or insomnia or parasomnias or nocturnal paroxysmal dystonia or rem sleep parasomnias or rem sleep behavior disorder* or sleep paralysis or sleep arousal disorder* or night terror* or somnambulism or sleep bruxism or sleep-wake transition disorder*).ab. /freq=2

50. exp mental health services/ or child guidance clinics/

51. exp mental health/

52. exp Child Guidance/

53. exp counseling/

54. (mental health service* or mental hygiene service* or mental health or counseling or motivational interview or pastoral care or child guidance).ti.

55. (mental health service* or mental hygiene service* or mental health or counseling or motivational interview or pastoral care or child guidance).ab. /freq=2

56. or/6-55

57. (adolescen* or young adult* or teen* or youth or emerging adult*).ti.

58. (adolescen* or young adult* or teen* or youth or emerging adult*).ab. /freq=2

59. 57 or 58

60. 5 and 56 and 59

61. limit 60 to (english language and yr="2004 -Current")

62. 5 and 56

63. limit 62 to (english language and (200 adolescence or 320 young adulthood ) and yr="2004 -Current")

64. (adolescen* or young adult* or teen* or youth or emerging adult*).tw.

65. 5 and 56 and 64

66. limit 65 to (english language and yr="2004 -Current")

67. limit 66 to (abstract collection or bibliography or chapter or clarification or "column/opinion" or "comment/reply" or dissertation or editorial or encyclopedia entry or "erratum/correction" or interview or letter or obituary or poetry or publication information or reprint or retraction or review-book or review-media or review-software & other or reviews)

68. 66 not 67
