# Supplementary material for: Primary care during the transition to adult care for adolescents involved with pediatric specialty services: a scoping review protocol
Source: Syst Rev. 2021 Feb 2;10:46. doi: 10.1186/s13643-021-01593-w (PMC7856752; doi:10.1186/s13643-021-01593-w)
Supplement: Supplementary file 3 — Additional file 3. Data Extraction Tool (Codebook). [file 13643_2021_1593_MOESM3_ESM.docx]

**Additional File 3.** Data Extraction Tool (Codebook)

| **Category** | **Data item** | **Item description** |
| --- | --- | --- |
| **Context** | 1. Country of origin | The country that the study was conducted in. |
|  | 2. Study type and aim | Describe study type and design, including relevant methodology. Describing the aim of the study. Where there are multiple aims, we will describe the main aim relevant to this study. Length of follow-up if available. |
|  | 3. Clinical setting and context | Description of clinical setting; unique features of context or how care is provided. Who do they serve (regional/geographical); types of patients (inpatient/outpatients); length of services provided within clinic or setting |
| **Population** | 4. Involved population/  participants | Participants and/or population involved  (Total N) |
|  | 5. Target diagnoses | Diagnoses (%s of population) of diagnoses, or conditions reported  If diagnoses not reported by clinic names are, report clinics  Note: if caregivers/clinicians were target population, then we put the name of diagnosis of AYAs they were discussing/served |
|  | 6. AYA age range | Mean, median, plus report any SD or IQR  *Note: this column will not be applicable to caregivers or clinicians, etc.  In brackets we will put time reported of age (may not need this).  If mean/median is not available (or only reported for large cohort); report % within a specified age range |
|  | 7. Other participant characteristics | Only use for very unique participant characteristics that we may want to capture |
| **Concept** | 8. Proportion of AYAs transferred to family physician | % of AYAs who followed by PCPs after specialist; or other model of care (transferred to specialist, co-care, etc.  If this is a calculated %, we will note this in the table – page # of article and how it was calculated |
|  | 9. Follow-up criteria | Description of eligibility criteria for who was transferred/followed by PCP |
|  | 10. Potential benefits of primary care involvement and/or relevant study findings | Outcomes associated with PCP involvement; relevant study findings about PCP role/involvement |
|  | 11. Knowledge/ beliefs and perceived facilitators/barriers about role of family physician | Only relevant for qualitative or mixed/multi methods studies.  This column will be blank for quantitative studies. This is not to be extracted from discussion or author opinion. |
|  | 12. Roles of Primary Care Physician (PCP) | Tasks or roles described as being the primary responsibility of the PCP. |
|  | 13. Model of care | Name or description of the model of care outlined in article. E.g., co-location co-mangament model, collaborative care model, integrated primary-specialist care model |
